# Characterising Parent-Appeal Marketing on Foods for Children: A Scoping Review

**DOI:** 10.1007/s13668-024-00559-3

**Published:** 2024-06-27

**Authors:** Alexandra Chung, Kostas Hatzikiriakidis, Florentine Martino, Helen Skouteris

**Affiliations:** 1 Department of Nutrition, Dietetics and Food, Monash University, Melbourne, Australia; 2 Health and Social Care Unit, School of Public Health and Preventive Medicine, Monash University, Melbourne, Australia; 3 Global Centre for Preventive Health and Nutrition, School of Health and Social Development, Faculty of Health, Institute for Health Transformation, Deakin University, Geelong, Australia; 4 Warwick Business School, Warwick University, Coventry, United Kingdom

**Keywords:** Food marketing, Parent-appeal, Child diets, Child nutrition

## Abstract

**Purpose of Review:**

This scoping review examines current evidence on parent-appeal marketing on the front-of-pack of food products for children and the impacts on parents’ perceptions, intentions, and behaviours.

**Recent Findings:**

Thirteen relevant studies were identified. Marketing features on packages of foods for children that appealed to parents include health claims, nutrition claims, non-nutrient claims such as 'natural', healthy-looking product images, images of healthy ingredients, and celebrity endorsements. At the same time, parents were wary of front-of-pack marketing and find it confusing, deceptive, and misleading. Child-appeal marketing features such as cartoon characters and bright colours gave parents the perception that products were unhealthy.

**Summary:**

Overall, this scoping review offers important insights into the types of front-of-pack marketing that appeal to parents and offers an inventory of parent-appeal marketing features. These findings support the design and implementation of policies that aim to reduce commercial influences on children’s diets through stronger regulation of marketing of foods for children.

**Supplementary Information:**

The online version contains supplementary material available at 10.1007/s13668-024-00559-3.

## Introduction

Marketing of food and non-alcoholic beverages negatively influences children’s dietary behaviours, increasing their consumption, choice, and preference for unhealthy food and drinks, contributing to poor diets and excess body weight [1]. Unhealthy food and beverage marketing is ubiquitous, present on television, in digital media, public spaces including around schools, in supermarkets, and on food packaging [2]. Foods designed for consumption by children are often heavily promoted through front-of-pack marketing that targets children and their caregivers, including colourful imagery, fictional characters and celebrities, appeals to fun, giveaways such as toys and collectible items, tie-ins with other brands, health claims promoting specific health effects of a product, and nutrition claims promote the presence or absence of certain nutrients in a product, [3, 4].

Despite the abundance of promotional imagery and messages on packages, many of the food products marketed for consumption by children fail to meet global nutrition standards [5]. For example, commercially available foods for infants and toddlers up to age 36 months, commonly contain high amounts of free sugars, despite being promoted as healthy and suitable for young children [6].

To reduce the impact of unhealthy food marketing on children’s diets, the World Health Organization (WHO) has urged governments to act to reduce children’s exposure to, and the impact of marketing of foods high in fats, sugars, and salt [7]. More recently, the WHO released policy guidance to protect children from the harmful impact of food marketing to support governments with policy design and implementation [8]. This much-needed guidance demonstrates increasing global attention on the need to regulate food marketing [9, 10]. Yet, policy guidance and much of the research to date, has focused on the nature of food marketing techniques that appeal to children. In contrast, limited research has focused on parent-appeal marketing, and subsequently, less is known about the nature or impact of front-of-pack marketing techniques targeting parents.

Evidence suggests that marketing on the packaging of food for children also targets parents [11]. This growing trend attracts parents’ attention at the point of sale and can influence the foods they purchase for their children [12, 13]. There is a need to better understand how parents are targeted with on-pack marketing on food and beverages for children (herein referred to as ‘food for children’) to build a more complete picture of how the food industry seeks to influences children’s diets. The objective of this scoping review was to map the current evidence on parent-appeal marketing on the front-of-pack of food for children.

Key questions informing the review were:

1. What types of front-of-pack marketing techniques appealing to parents on children’s food and beverage packages have been examined in the literature?
2. What are the impacts of front-of-pack marketing techniques on parent and caregivers’ product perceptions, intentions, and behaviours?

## Methods

A preliminary search of MEDLINE and the Cochrane Database of Systematic Reviews found no systematic reviews or scoping reviews on the topic of parent-appeal marketing. A scoping review was therefore identified as the approach for this study to identify and map the existing body of academic literature in this field [14]. This scoping review was guided by the JBI’s methodological guidance for conducting scoping reviews [15] and reported in accordance with the Preferred Reporting Items for Systematic Reviews and Meta-Analyses Extension for Scoping Reviews (PRISMA-ScR) [16]. This review was registered prospectively with Open Science Framework Registries: [10.17605/OSF.IO/M3RXP].

A systematic search of four electronic health and public health databases (Ovid MEDLINE, Embase, Web of Science, Global Health (EBSCO), was conducted in September 2022. Search terms were identified for each of the following concepts: ‘marketing’, ‘parents’, and ‘food and beverages’ and applied to title, abstract, and keyword searches (Supplementary Table 1). Subject headings were used where applicable, and the search strategy was adapted for each database. Date limits were set from 2010-current. Reference lists of all included articles were scanned for additional relevant studies. Data searching, screening, and extraction was supported using Endnote citation manager and Covidence systematic review software.

Experimental, observational, and descriptive studies published in English were included if they reported on: 1) The types of marketing techniques on the front-of-pack of foods for children that appeal to parents of children up to 18 years of age; and 2) The impact of marketing techniques used on the front-of-pack of foods for children on parents' purchase intentions and behaviours that relate to feeding their child(ren). Studies that reported on marketing across media other than front-of-pack (television, digital, point of sale, sponsorship etc.) and studies that reported exclusively on the marketing of commercial infant formula / breast milk substitutes were excluded.

The results of the search were exported to Covidence for screening. All records were screened against the inclusion criteria at the title and abstract stage by a single author (AC, KH, or FM). If it was not clear whether a study should be included from reading the title and abstract, the full paper was assessed for eligibility. Full text records were then reviewed independently by two authors (AC and KH or FM) with authors meeting during the process to discuss any discrepancies. The reference lists of included records were then screened for additional eligible articles. The search procedure was repeated in June 2024 to identify any eligible studies published since the initial database search. 

Data extraction was completed by a single author (AC) and cross-checked for accuracy by a second author (KH). Data items included: study aim; participants; sample size; study design and methods; marketing features examined; food items examined; results; key findings relevant to the review questions. Narrative synthesis was then undertaken to describe key findings as relevant to the aims of this review.

## Results

Initial database searches identified a total of 13,105 records. Following removal of 4,334 duplicates, the titles and abstracts of 8,771 unique records were screened. At the full-text stage, 100 articles were screened against the inclusion criteria, and a total of eight articles were considered eligible for inclusion. During this stage, one additional record was identified via the reference list of eligible studies. During the updated search a further four eligible studies were identified. For study selection flow diagram see Supplementary Fig. 1. Thirteen articles were therefore identified for inclusion in this review [17–28], published between 2011 and 2024 (Supplementary Table 2). Six studies were undertaken in the United States, five in Australia, and one each in Mexico and Uruguay.

### Research question 1

What types of front-of-pack marketing techniques appealing to parents on children’s food and beverage packages have been examined in the literature?

Studies identified in this review examined at front-of-pack marketing features such as: health claims, nutrient claims, non-nutrient claims, celebrity endorsements, images of the product, images of healthy ingredients within the product, cartoon characters and other child-appealing colours. Of note, some marketing techniques were defined differently across studies. For example, several studies used the term health claims to refer to claims pertaining to health benefits as well as claims around specific nutrients. For this study, we defined ‘health claims’ as those which make an assertion about the benefit of a product or component of a product on physical health; ‘nutrient claims’ as those promoting the presence or absence of a nutrient in a product, and; 'non-nutrient claims’ as those that describe a desirable feature of the product or its ingredients such as ‘natural’, ‘organic’, and ‘no preservatives’ [29].

Studies explored marketing present on the front-of-pack of food products, including breakfast cereals; packaged snacks such as cereal bars, fruit snacks, and cheese dip snacks; yoghurt; ice cream bars; cakes; chicken nuggets; flavoured milk and sweetened fruit drinks.

### Research question 2:

What are the impacts of front-of-pack marketing techniques on parents' product perceptions, intentions and behaviours?

Several marketing features were reported to appeal to parents, impacting parents in ways that elicited positive product associations or encouraged product selection or purchase (Table 1.) Studies examined the effect of front-of-pack marketing features on parents’ perceptions of healthiness of a product [17, 19–23, 25]; product preferences or choices [19, 20, 23, 24]; and purchase intent or willingness to buy [18, 21].

**Table 1:** Inventory of front-of-pack marketing features that appeal to parents

**Marketing techniques that appeal to parents**	**Examples**
Health claims	Supports your child's immunity
Nutrition claims	Source of fibre; source of calcium; good source of omega 3; no added sugar; no added salt; made with real fruit; whole grain
Non-nutrient claims	Natural; no preservatives, colours or flavours; organic
Images of the product	Healthy-looking image of product; natural-looking products (e.g., yellow cereal consistent with corn-flakes)
Visuals of healthy ingredients	Images of fresh fruit
Celebrity endorsements	Image of popular sportsperson with attributed quote including nutrient claim and positive product attributes (e.g., taste, convenience)

Nutrient claims were the most common marketing feature examined across the included studies. Claims promoting the presence of healthy or desirable nutrients in products (source of calcium and vitamin D, full serving of fruit) were shown to positively influence parents’ perceptions [17, 19–21] and choices or willingness to buy [21–25, 28]. Claims promoting the absence of unhealthy or undesirable ingredients, also called 'free from' claims (no added sugar, no preservatives) also influenced parents in favour of those products [17, 19, 20, 22, 23, 26, 28, 30].

Non-nutrient claims, such as ‘natural’, ‘no preservatives’, and ‘organic’, were found to influence parents, creating the perception that products are healthy [17, 20, 21, 23, 27, 30]. Front-of-pack images that indicated a natural-looking product were favoured by parents over products that appeared to be artificially coloured or flavoured or chocolate-based [17, 24]. Visuals of healthy ingredients also played a significant role in creating perceptions of healthiness and motivating parents to opt for these products [17, 20].

Only one study examined the use of health claims on food packages. Harris et al. tested the effects of the health claim ‘supports your child’s immunity’ on cereal boxes and found that this led most parents (75%) to believe that the product might keep their child from getting sick [21].

The presence of celebrity endorsements were examined in one study, with results demonstrating that the visual of a sports celebrity alongside a relevant quote about the product’s nutrition, taste, and convenience had a positive impact on parents’ preferences towards the product [19].

Marketing features that typically appeal to children including cartoon characters, colourful packaging and playful visuals were found to contribute to unhealthy product associations but did not influence parent behaviour in the studies examined in this review (Table 2). Parents associated cartoon characters and playful visuals on packaged fruit snacks with high sugar content and artificial ingredients [17, 23]. On cereal packages, the presence of cartoon characters gave the impression that the product would not be suitable for children [18]. In contrast, two studies found that the presence of cartoon characters had little [25] or no impact on parent choices [24].

**Table 2:** Inventory of front-of-pack marketing features that elicit negative or unhealthy product associations among parents

**Marketing techniques that elicit negative or unhealthy product associations**	**Examples**
Cartoon characters	Licenced characters, generic cartoon characters
Fun or playful visuals	Playful shapes, playful characters
Colourful packaging	Bright colours on packaging

When asked about their perceptions of front-of-pack marketing, parents reported that front-of-pack marketing is misleading, deceptive, and confusing [17, 20]. Nevertheless, in one of these studies, parents acknowledged that they rarely question their first positive impression and are influenced by the marketing messages when making food choices [17].

## Discussion and Implications

This scoping review synthesised evidence from thirteen studies to describe the impacts of front-of-pack marketing on foods for children on parents’ perceptions and purchasing behaviours. Front-of-pack marketing, including images of fruits, claims about health benefits, nutrient claims promoting the presence of healthy or desirable nutrients, and the absence of undesirable nutrients, and claims about the product being 'natural', had a positive impact on parents’ perceptions and choices. On the other hand, parents perceived products featuring children's cartoon characters on the front-of-pack as being less healthy for children.

Health and nutrition claims are common on food packaging, often added to products at the discretion of manufacturers. The use of front-of-pack claims creates a health-halo effect by implying that products are healthy, or healthier than similar competitor products [31]. Findings from the current review show that nutrient claims on food for children increased perceptions of health and influenced parents’ choices and intent to buy. This is echoed in other research on packaged foods that has found nutrition claims influence consumer perceptions and behaviours. For example, on-pack claims promoting the presence of desirable nutrients such as protein, fibre, calcium, and vitamin C, and the absence of undesirable nutrients, such as saturated fat and sodium, positively influenced consumer perceptions of a product’s healthiness [30, 32]. Reviews of the impact of claims on packaged foods have found that the presence of health and nutrition claims influence purchase intentions, and purchase and consumption behaviours among adults [33, 34]. Research testing consumer responses to non-nutrient claims, such as ‘organic’ and ‘natural’, on food packages similarly found a positive effect on the perceived healthiness of products and intention to purchase [30, 35].

Findings of this review indicate that cartoon characters, colourful packaging, and playful visuals on food for children create the perception among parents that products are unhealthy. These features are prominent on food for children and are considered forms of child-directed marketing [3]. It is worth noting that whilst these marketing features may imply a less healthy product, they did not necessarily deter parents from making a purchase.

This review found that parents reported front-of-pack marketing as confusing, deceptive, and misleading. Other research has demonstrated the ways in which claims on food packages are misleading consumers. For example health-related claims are often present on non-core or discretionary foods [36], and ‘free from sugar’ claims are frequently observed on infant and toddler food packages, despite these foods commonly sweetened with free sugars in the form of fruit juice, powder, or purees [31, 37]. This highlights the need for stronger regulation around the use of health, nutrition, and non-nutrient claims, and more transparent labelling of added sugars in foods.

Findings demonstrate that front-of-pack marketing remains an important area for monitoring and regulation. This study characterises the nature of parent-appeal marketing and shows that front-of-pack marketing impacts parents’ perceptions and choices of foods for their children. Many of the marketing techniques present on food for children appeal to parents and are not considered child-appealing [4]. This could have important implications for the design of policy reform to reduce the harmful impacts of food marketing. Should legislation solely focus on child-appealing marketing techniques, in line with existing recommendations [10], parent-appealing marketing features may be missed. This oversight could create a loophole, allowing the food industry to continue influencing children’s diets. Experts have recommended key considerations for legislative measures to protect children’s diets from unhealthy food marketing, including expanding the focus from 'child-directed' marketing to all marketing that children up to age 18 are exposed to; designing tailored regulations for multiple settings, media, and marketing techniques; strengthening underlying food classification systems; and strengthening monitoring and enforcement systems [38]. This review provides evidence to extend these recommendations to restrict front-of-pack marketing that appeals to parents.

## Limitations

Only a small number of studies were found to report on parent-appeal marketing on foods for children. Of those, only a small number of food products were examined – most commonly breakfast cereals, often in experimental settings. This highlights the need for ongoing research to monitor parent-appeal marketing on food for children across a wider range of food products and in real-world contexts. Further research is also needed to identify other ways in which parents are targeted with food marketing. This study examined literature in English only. Exploration of non-English literature may reveal further insights.

## Conclusion

Parents are being deliberately targeted by the food industry through front-of-pack marketing. Parents are sceptical about the intent of front-of-pack marketing, but at the same time are vulnerable to the impacts of persuasive marketing techniques. This scoping review uses available evidence to build an inventory of parent-appeal marketing and provides a deeper understanding of the types of front-of-pack marketing techniques that target parents on foods for children. This study provides a framework for further research across a wider range of food products and in real-world contexts to grow the evidence base and support policy change. These findings will enable ongoing monitoring of marketing practices that target parents and inform advocacy and comprehensive policy design to protect children’s diets from commercial interests.

### Supplementary Information

Below is the link to the electronic supplementary material.

Supplementary file1 (DOCX 60.5 KB)
