# Supplementary material for: Characterising Parent-Appeal Marketing on Foods for Children: A Scoping Review
Source: Curr Nutr Rep. 2024 Jun 27;13(3):393–8. doi: 10.1007/s13668-024-00559-3 (PMC11327212; doi:10.1007/s13668-024-00559-3)
Supplement: Supplementary file 1 — Supplementary file1 (DOCX 60.5 KB) [file 13668_2024_559_MOESM1_ESM.docx]

**Supplementary Material**

Title: Characterising parent-appeal marketing on foods for children: A scoping review.

Journal: Current Nutrition Reports

Authors: Alexandra Chung, Kostas Hatzikirikiadis, Florentine Martino, Helen Skouteris

Corresponding author: Alexandra Chung. Department of Nutrition, Dietetics and Food, Monash University, Melbourne Australia

Email: Alexandra.chung@monash.edu

***Supplementary Table 1****. Search terms*

| **Topic 1: marketing (combine the below terms with “OR”)** | **Topic 2: Parents (combine the below terms with “OR”)** | **Topic 3: Food and beverage (combine the below terms with “OR”)** |
| --- | --- | --- |
| “marketing” | “parent” | Exp food/ |
| “advertis*” | “mother” | Exp beverages/ |
| “advertiz*” | “father” | Exp diet/ |
| “packaging” | “caregiver” |  |
| “labels” | “carer” |  |
| “labelling” | “family” |  |
| “promotion” | “families” |  |
| “branding” | “child” |  |
|  | “infant” |  |
|  | “toddler” |  |
|  | “adolescen*” |  |
|  | “teenage” |  |

“/” denotes subject heading (Mesh term)

**Identification of included studies**

Duplicate records removed

(n = 4334)

Records identified from databases (n = 13,105)

**Identification**

Records screened

(n = 8771)

Records excluded

(n = 8671)

**Screening**

Reports excluded:

Not FOP marketing (n = 27)

Not parents/caregivers (n = 22)

Not primary research (n = 21)

Not food for children (n = 19)

Not parent-appeal (n = 3)

Reports assessed for eligibility

(n = 100)

Reports identified from reference list of eligible studies

(n = 1)

Reports identified from updated search

(n = 4)

Studies included in review

(n = 13)

**Included**

**Supplementary Figure 1.** Study selection flow diagram

***Supplementary Table 2****. Characteristics of included studies*

| Study | Study design | Marketing features examined | Food products examined | Key findings |
| --- | --- | --- | --- | --- |
| Abrams et al (2014). Ignorance is bliss. How parents of preschool children make sense of front-of-pack visuals and claims on food. | Focus groups | Front-of-pack visuals and nutrition and non-nutrient claims | Packaged fruit snacks (actual packages and mock packages) | Realistic visuals of fruit, product images, nutrition claims (fat free, no sugar added, full serving of fruit), non-nutrient claims (it, no artificial colours, flavours, preservatives), and visuals suggesting the product is more natural led parents to believe a product is healthier. Characters and bright colours on-pack were perceived as ‘unhealthy’ by parents (higher sugar, artificial ingredients, fewer health benefits). While parents recognized that the health claims and some visuals may not necessarily indicate that the food is healthier, they reported that they rarely think beyond their initial positive impression. |
| Contreras-Manzano et al (2020). The impact of a cartoon character on adults perceptions of children’s breakfast cereals. | Online experimental study | Cartoon characters | Breakfast cereal | Cartoon characters on pack were associated with parents’ perception that the cereal was ‘not a good product’ to buy for children |
| Dixon et al (2011). Parents’ responses to nutrient claims and sports celebrity endorsements on energy dense and nutrient poor foods: an experimental study | Online experimental study | Nutrient claims and sports celebrity endorsements | Sweetened breakfast cereals; cheese dip snacks; ice cream bars; frozen chicken nuggets; and flavoured milk drinks | Nutrient claims (source of fibre, source of calcium, reduced fat, trans fat free, good source of vitamin D) influenced parents’ preferences (towards these products) among the majority who do not read the nutrition information panel. The effect of nutrient claims were negated among parents who read the nutrition information panel. Sports celebrity endorsements (visual and text corresponding to the nutrient claim e.g., a tasty source of fibre to start your kid’s day) positively influenced parent preferences. |
| Dixon et al (2024). Effects of marketing claims on toddler food products on parents' product preferences, perceptions and purchasing intentions: an online experiment | Online experimental study | Nutrition claims, non-nutrient claims | Banana bars, strawberry snacks, blueberry yogurt snacks and veggie snacks | 'Free from’ claims (preservative free; nothing artificial) increased participant's intentions to purchase unhealthy food products for their toddlers. 'Contains good’ claims (made with whole grains; contains real vegetables) and 'child-related' claims (encourages self-feeding) showed limited effects on parent's preferences, purchase intentions and perceptions. |
| Fleming-Milici et al (2022). Marketing of sugar sweetened children’s drinks and parents’ misperceptions about benefits for young children. | Focus groups | Front-of-pack visuals, nutrition claims, product names | Sugar-sweetened children's drinks, including sweetened fruit-flavoured drinks and toddler milks | Nutrition claims (vitamin C, less sugar), non-nutrient claims (100% natural) and visuals of healthy ingredients (fruit) gave the perception that the product was healthy for children and encouraged parents to choose those products for their child. Parents perceived front-of-pack claims, images, and marketing messages as confusing, deceptive, and misleading. |
| Hall et al (2023). Natural Claims on Sugary Fruit Drinks: A Randomized Experiment With U.S. Parents | Online experimental study | Non-nutrient claims | Fruit drinks | Non-nutrient claims (natural, 100% all natural) led parents to have higher intentions to purchase a fruit drink for their child. The natural claim also led parents to think that the fruit drink was healthier for their children. The claims made parents less likely to think that the drink contained added sugar and estimate lower added sugar content. Mediation analyses revealed that the claims led to higher intentions to purchase the fruit drink by increasing perceived healthfulness of the fruit drink and by leading parents to believe that there was no added sugar in the fruit drink. |
| Hall et al (2022). Nutrition-related claims lead parents to choose less healthy drinks for young children: a randomized trial in a virtual convenience store | Online experimental study (virtual convenience store) | Nutrition claims, non-nutrient claims | Fruit drinks | Nutrition (100% vitamin C) and non-nutrient ("No artificial sweeteners" and "100% All Natural") claims on fruit drinks increased the likelihood that parents selected the fruit drink instead of fruit juice. Non-nutrient claims increased the likelihood of parents choosing the fruit drink instead of water, but the "100% Vitamin C" nutrient claim did not. All claims made parents more likely to incorrectly believe that the fruit drinks contained no added sugar and were 100% juice. |
| Harris et al (2011). Nutrition-related claims on children’s cereals: What do they mean to parents and do they influence willingness to buy? | Online survey | Health claims, nutrition claims, non-nutrient claims | Children’s breakfast cereals (of below average nutritional quality) | Health claims (supports your child’s immunity) led to the belief that the product might keep their child from getting sick. Health claims, nutrition content claims (whole grain, fibre, Good source of calcium & vitamin D) and non-nutrient claims (organic) gave parents the sense that products are more nutritious overall. Nutrition content claims gave the perception that products might provide specific health benefits for children which increased parents' willingness to buy these cereals. |
| McCann et al (2022). Regulated nutrition claims increase perceived healthiness of an ultra-processed, discretionary toddler snack food and ultra-processed toddler milks: a discrete choice experiment. | Discrete choice experiment | Nutrition claims, non-nutrient claims | Cereal bar, toddler milk | Nutrition claims (no added sugar, no added salt, good source of omega 3) increased perceptions of product healthiness (compared to no claims). Non-nutrient claims (no added preservatives, colours or flavours, made with real fruit, organic) increased perceptions of product healthiness. |
| Musicus et al (2022). Effect of Front-of-Package Information, Fruit Imagery, and High-Added Sugar Warning Labels on Parent Beverage Choices for Children: A Randomized Clinical Trial | Online experimental study | Nutrition claims, fruit imagery | Fruit drinks | Removing a 100% vitamin C claim and fruit imagery resulted in fewer parents selecting high–added sugar beverages for their children. Removing only the 100% vitamin C claim, or the fruit imagery had no behavioral effects independently. Claim and imagery removal had no knowledge or health perception effects combined or independently. |
| Pulker et al (2019). The impact of voluntary policies on parents' ability to select healthy foods in supermarkets: A qualitative study of Australian parental views. | Focus groups | Front-of-pack cartoon characters, unusual shapes and bright colours, nutrition claims, non-nutrient claims | Breakfast cereal, cereal bars, packaged snacks sweet and savoury, kids' yoghurts | Nutrition claims (low sugar, wholegrain) and non-nutrient claims (no preservatives) positively influenced parents’ choices. Child-appealing colours, characters were perceived to be associated with unhealthy / high sugar products. Food manufacturers’ messages were not trusted by parents. |
| Russell et al (2017). The impact of front of pack marketing attributes versus nutrition and health information on parents’ food choices | Discrete choice experiment | Nutrition claims, product visuals (bowl with cereal), additional visuals (wholegrains; fresh fruit; cartoon puppy; cartoon sports kid) | Breakfast cereal | Product visuals were found to be the most significant attributes in driving parents’ choices. Parents preferred visuals of neutral cereals (bran) and disliked cereals with artificial and chocolate looking cereals. Fresh fruit visuals and nutrient claims (good source of folate, iron, and vitamins) positively influenced choices. Cartoon characters had little effect on parents’ choices. |
| Velazquez et al (2021). The influence of label information on the snacks parents choose for their children: individual differences in a choice based conjoint test. | Choice-conjoint experiment | Nutrition claims, cartoon characters | Sponge cake and chocolate milk | Nutrition claims (source of calcium and vitamin D, with all the fibre of cereals) positively influenced mothers’ choices. Nutrition claims had higher importance than objective health information on NIP.  Cartoon characters had low impact on mothers’ choice and healthiness perception. |
